# Suppurative infections after lower third molar surgery: a systematic review

**DOI:** 10.4317/medoral.27213

**Published:** 2025-08-16

**Authors:** Roberto Pippi, Umberto Giuliani

**Affiliations:** 1 pienza University of Rome, Department of Odontostomatological and Maxillo Facial Sciences

## Abstract

**Background:**

After lower third molar surgery, suppurative infections can occur. They can spread into adjacent tissues, toward superficial mucosal or skin planes or toward deep facial and neck fascial spaces.

**Material and Methods:**

The 2020 PRISMA guidelines for systematic reviews were followed. A literature search was conducted, without initial time limit, in the Medline database, via Pubmed (MEDLINE), and SCOPUS. For the analysis of statistical significance, the hypothesis test on the difference between means with unknown variances was used.

**Results:**

Eleven articles met the inclusion criteria. The total number of extractions taken from the articles included in the review is 7363, with a 5.35% total incidence of purulent infections. The incidence of purulent infections was found significantly higher in cases in which antibiotic prophylaxis was not performed.

**Conclusions:**

Although lower third molar extraction is the most frequently performed oral surgical procedure, little has been written about post-operative purulent infections, in relation to risk factors, involved spaces, and performed therapies, so to be able to identify preventive and therapeutic behaviors based on scientific evidence. Antibiotic prophylaxis is the only variable which resulted in determining a significative statistical reduction in the incidence of purulent infection after lower third molar surgery.

** Key words:**Antibiotic prophylaxis, risk factors, ostectomy, involved spaces.

## Introduction

Extraction of lower third molars is one of the most common oral surgical interventions (1). Like other surgeries, surgeons must deal with post-operative complications, such as pain, trismus, edema, temporary or permanent neurological damage and infections (2) which include wound infections such as alveolar osteitis and alveolar abscesses, also late mucoperiosteal abscesses. Abscesses are suppurative infections (SIs) which can spread into adjacent tissues, toward superficial mucosal or skin planes or toward deep facial and neck fascial spaces (3) and only sometimes spontaneously open towards mucosal or skin surfaces with pus discharge. In the development of SIs many microbial and patient factors are involved, including the type, site, size, and depth of the wound, the possible contamination by exogenous material, the extent of wound blood perfusion, the general health and immune status of the patient, the microbial load, and the virulence of the involved microorganisms. Moreover, since third molar surgery is not a clean operative procedure, surgical SIs usually have a polymicrobial etiology, involving both aerobic and anaerobic microorganisms (4,5).

-Why it is important to do this review

Suppurative infections are high-impact events that may expose patients to severe and sometimes life-threatening complications, often forcing clinicians to perform an empirical choice, preventive (6) or treatment (7), related to their personal experience, without substantial scientific support. Moreover, the awareness of general dental practitioners and oral surgeons regarding the actual incidence of these infections and their risk factors may reduce malpractice and incorrect surgical decisions, as well as medico-legal litigations, helping clinicians to acquire informed consent from patients. Finally, since third molar surgeries performed by general dental practitioners were found to be more frequently affected by serious SIs than those performed by oral surgeons (8), the perfect knowledge of related problems is necessary to allow general dental practitioners to accurately assess what surgeries should be preferably referred to an oral surgeon.

The main aim of the present work is to analyze the incidence of SIs after surgical extraction of lower third molars. The secondary aim is to investigate the existence of possible risk factors related to the occurrence and gravity of this kind of infections.

## Material and Methods

The 2020 PRISMA guidelines for systematic reviews were followed. The present review was not registered on any international platform for systematic reviews and the review protocol was not prepared. The two researchers conducted a literature search independently, without an initial time limit, in the Medline database, via Pubmed (MEDLINE), and SCOPUS. The results of each search were then compared to verify the procedure. For Pubmed, the search strategy used a combination of the controlled vocabulary, MeSH terms, with free text terms. The search string has been inserted in the "advanced search" section. The total number of items generated is the result of the following two combinations: - (((((abscess [MeSH Terms]) OR (abscess)) OR ((infection [MeSH Terms]) OR (infection))) OR ((suppuration [MeSH Terms]) OR (suppuration))) OR ( (phlegmon [MeSH Terms]) OR (phlegmon))) OR ((cellulitis [MeSH Terms]) OR (cellulitis))- ((tooth extraction [MeSH Terms]) OR (tooth extraction)) OR ((tooth avulsion [MeSH Terms]) OR (tooth avulsion)).

For SCOPUS, the search was conducted by inserting the terms “third molar extraction and infection” and then “third molar extraction and post-operative complications” into the search string, with the aim of including the greatest possible number of articles.

The results of the above search process have been merged, filtered (Filters: Humans, English), and duplicates have been removed. A manual search was also performed on the references of the articles identified by the electronic search.

Randomized controlled trials, prospective studies, retrospective studies, and case series were considered. Only studies that met the following inclusion criteria were selected for the following review:

1. studies in English

2. studies conducted on humans

3. studies in which SIs occurred after lower third molar surgical extraction

 The following criteria were followed for an article to be excluded:

1. *in vitro* studies

2. non-English Language studies

3. endodontic abscesses or pre-extraction abscesses

4. studies on abscesses or infections that were not post-extraction infections

5. extraction of teeth other than the lower third molar

6. non-surgical extractions

7. non-suppurative infections

In all collected studies, the following data were searched for: the number of enrolled patients, the number of SIs found, the number of cases with and without antibiotic prophylaxis (AP), antibiotic drugs used for AP, the number of cases with and without ostectomy, any other possible risk factors, the number of cases with hospitalization, sites of infection, and the kind of treatment performed for infection.

For the analysis of statistical significance, the Welch hypothesis test on the difference between means with unknown variances was used. This test is a variant of the Student t-test which is used to compare the means of two independent groups, when the assumptions of the classical t-test are not satisfied, in particular when the variances of the two groups are different (heteroscedasticity). Moreover, this test is more robust than the Student t-test in situations where the variances are not equal. Since with an alpha=0.05, the extremes of the Gaussian curve are located at ± 1.64, the comparison between the means predicts statistical significance when the Z values ​​are located outside the extremes of the curve.

Two variables, that is “Antibiotic Prophylaxis” (AP) and “Ostectomy”, were investigated about the incidence of SIs in the study population.

## Results

**Figure 1:**
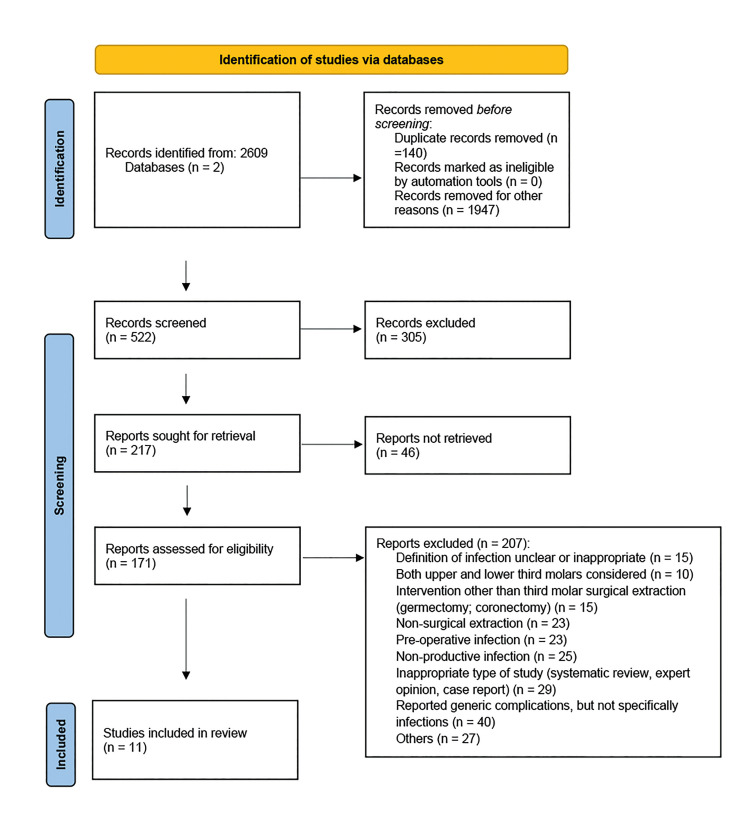
2020 PRISMA Statement. Flow-chart.

After the search, no specific trials were found about the risk of SIs after lower third molar surgery; therefore, data concerning study outcomes were extracted from studies performed with other aims. From the initial 191 studies retrieved, 11 articles met the inclusion criteria (Fig. 1, Table 1), while 180 were excluded for several reasons.

The risk of bias in the present study is high because data were extrapolated from different kinds of studies to find information about each specific study outcome and are possibly related to reporting or missing data, inadequate detection, missing variables, and misclassification.

The total number of extractions taken from the articles included in the review is 7363, with a total incidence of SIs of 4.34 % (n = 320).

Regarding the incidence of SIs in relation to some variables, it is not possible to obtain a single Figure as some studies consider the patient as the statistical unit, (9,10) others the single tooth extraction, (11-13) while in only four studies the number of extractions coincides with the number of patients (14-18).

Considering the number of patients, SIs incidence for those who have had antibiotic prophylaxis (AP; *n* = 1,644) is 1.33% while for those who have no AP (n = 731) it is 6.56%. If the number of extractions is considered, SIs incidence for extractions carried out under AP is 3.62% (n = 5,106), while for those carried out without AP it is 4.8% (n = 1,603). The difference between these incidences is statistically significant (both for patients: Z = ± 21.7 and extractions: Z = ± 2.97 - Table 2).

Considering the studies in which the number of extractions coincides with the number of patients, SIs incidence for the AP group is 1.36% (n = 1464), while for the no AP group (n = 414) the incidence is 6.82%. The difference is statistically significant (Z = ± 18.3).

Considering ostectomy, SIs incidence in patients in whom it was performed is 4% (n = 873), while in those in whom it was not performed SIs incidence is 3.47% (n = 115). Regarding the extractions, SIs incidence is 3.92% for those in which ostectomy was performed (n = 1014), while it is 3.45% for those in which it was not (n = 115). The difference between these incidences is not statistically significant (both for patients: Z = ± 0.86, and extractions: Z = ± 0.8).

Considering the studies in which the number of extractions coincides with the number of patients, SIs incidence for the Ostectomy group (n = 693) is 3.3% (n = 23), while the incidence for the no Ostectomy group (n = 115) is 3.48% (n = 4). The difference is not statistically significant (Z = ± 0,07).

Of the total number of SIs (n. = 320), 8 developed in the submandibular space, 1 in the sublingual space, 1 in the vestibule, 1 in the parapharyngeal space. Two cases of intraoral abscess, whose site was not specified, and 1 case of a deep wound infection that was not better specified were reported. Of the remaining cases, it is not known if and where an infection spreading was present.

Most of the selected studies do not provide information about patients' risk factors (10-15). In one of these studies, patients with systemic risk factors were even excluded (17), while in another (9), patients who developed infections had no systemic risk factors. In the study of Goldberg *et al*. (16), out of the 21 patients with infections, 20 were inpatients due to their systemic conditions (diabetes mellitus or corticosteroid therapy) or to the difficulty of surgery. Unfortunately, it is not clear how many of the 125 inpatients were hospitalized for their systemic conditions. In the study of Yoshii *et al*. (19), however, 3.5% of patients with compromised systemic conditions had SIs, but only one of them was immunosuppressed by corticosteroid therapy. The same study underlined that there were no differences in the incidence of infections between healthy patients and medically compromised patients. However, no study mentioned the presence of local risk factors and/or the occurrence of intraoperative accidents. Two of the selected studies analyzed the correlation with AP and ostectomy (13,14).

Out of the total number of infections, only 12 patients were hospitalized. Not all selected studies specified the therapies for SIs. The following modalities were reported: antibiotic therapy at home (15,16); incision and drainage of the infection (9-11); antibiotic therapy during hospitalization (9).

## Discussion

A great difficulty in drafting the present review concerned the definition of SI. Actually, many studies, whose full texts were analyzed, were not included in the final qualitative analysis due to the absence of a definition of "infection". The present research was very stringent in this regard: in fact, only studies in which there were, or it was implied that there were suppurative, fluctuating infections, possibly extended to the neighboring anatomical areas (e.g. lodges, fascial spaces, intermuscular spaces), were included. Where the definition of infection was not clear, thus including the possibility that it was an alveolar osteitis, the article was considered not eligible as these infections were excluded from the setting criteria of the study (20-24).

The incidence of SIs after lower third molar surgery is not unique in the international literature (0,75% (9), 0,8% (10) -16% (13)), given the extreme variability of the studies in this regard both in terms of sample size and approach, so a real estimate of the problem is not possible. Most times, data are findings of studies focused on other variables analysis. Therefore, it is not possible to estimate a single incidence datum. Moreover, the data is underestimated because third molar extraction is a surgical procedure mostly performed in a private setting and therefore not brought to the attention of the scientific world through publication in specialized journals. The same is true for extractions performed in a hospital/university setting as not all of them are the subject of research and therefore of publication. The incidence of SIs after lower third molar surgery is therefore underestimated, and this can also negatively influence the discussion with patients about the prophylactic removal of asymptomatic lower third molars (8).

It is even more evident the lack of descriptive details relating to the infection type, especially in cases of infections that spread into deep spaces such as fascia and lodges. In most studies, when infection is mentioned, it is detected as a secondary finding and not directly investigated as a study variable (9,11,16). On the contrary, where the infection is the study variable, with all its sequelae, many data are available, but all concerning surgery, and those on preoperative prophylaxis are still lacking (25). The only descriptive details on the infectious sequelae and their treatment, not always linked to the surgical extractions of the lower third molars, can be found in several case reports (26-28), just to confirm the exceptionality of the event. It is therefore possible to conclude that postoperative infection of third molar surgery as a study variable is not very attractive to international research groups, perhaps due to its low incidence.

In one of the selected articles (9), authors excluded cases in which post-operative infections occurred, and which instead have been considered for the present review since their data were available. On the other hand, if in other studies post-operative infections occurred as well, and the relative cases have been excluded, relative data are possibly not be available for review. Furthermore, many studies, albeit report the incidence of infections, consider both upper and lower third molars, making the selective extraction of data for the ones or the others not possible (29,30). These studies were therefore not considered eligible for the present review.

Undoubtedly the fact that an adequate antibiotic therapy alone is often the only solution for SIs, when they do not spread to neighboring and deep spaces, may justify a lower interest from the scientific world but does not allow a correct evaluation of the extent and severity of the phenomenon, nor allow surgeons to be able to provide correct information to the patient before extraction, and, lastly, it increases the amount of antibiotics taken worldwide, increasing both bacterial resistance and adverse reactions to antibiotics (31).

The only variable which resulted in determining a significative statistical difference in the incidence of infection is AP (Table 2). Actually, in the subgroups in which AP, considered as the only variable, was given, the incidence of infections was lower than in the subgroups in which prophylaxis was not given. This result agrees with data from the most recent Cochrane review about AP for third molar surgery, which found that, regardless of the antibiotic used, antibiotics may reduce infections by approximately 66% (RR 0.34), although as many as 19 people (95% CI) need to be treated with antibiotics to prevent one infection. (6).

In line with the results of the present review, the only two studies which linked the two variables "ostectomy" and "antibiotic prophylaxis"(13,14) found that the subgroups in which ostectomy was performed, and AP was not performed had a higher incidence of post-operative infections, despite the differences in the incidence of infection between cases with and those without ostectomy were not statistically significant. Therefore, the inverse association of the two variables was associated with a higher incidence rate.

As for the different drugs used for antibiotic prophylaxis, in the studies performed in the last 30 years (10,14,15,18) amoxicillin (with or without clavulanic acid) alone or associated with the metronidazole were mostly used. This is related to the usual mixed etiology of SIs, with both aerobic and anaerobic microorganisms (4), since amoxicillin is the most indicated antibiotic for oral aerobic microorganisms and metronidazole is most indicated for anaerobes.

Unfortunately, only a few studies reported if and where the infection spread, while the site of spreading and the number of spaces involved have been considered important factors in determining the severity of an infection, in relation to the patient’s life risk. Indeed, Flynn and Shanti (32) suggested a severity score for odontogenic infections, attributing from 1 to 3 points for each space, based on their proximity to vital anatomic spaces, such as airways and mediastinum, with a final score that was the result of the sum of all scores, if more than one space was involved. Actually, albeit sporadically, these infections may represent medical-surgical emergencies, which must be treated timely.

Many systemic conditions, including diabetes mellitus, alcohol, cigarette smoking, chronic immunosuppression, chemotherapy and radiotherapy (33), have been considered trigger factors for spreading or worsening oral SIs. In the study of Hasegawa *et al*. (34), diabetic patients were exposed to a risk of developing odontogenic infections like that of healthy patients, but with a greater ability to spread into the fascial spaces and neck.

Despite this, from the selected articles, it is not possible to identify whether immunosuppression, linked to compromised medical conditions, may or may not represent a risk factor for the occurrence of SIs after third molar extraction. Likewise, no local factors are reported in relation to such infections. On the other hand, the study of Berge (8) reported a high rate of both pre-operative infection (53,85%) and smoker status (53,85%) in cases which required hospitalization after third molar extraction and in which the relative data were available (15/22). These rates, although referred to a small sample, suggest considering preoperative infection and smoking as risk factors for post-operative SIs after lower third molar surgery (8).

Hospitalization was rarely performed but it was in all cases in which the infection involved 1 or more deep fascial spaces (buccal, submandibular, lateral-pharyngeal, sublingual, para-pharyngeal) (9,13,16). Criteria have been proposed for odontogenic infections to determine whether a patient needs hospitalization (35) (Table 3), although incessant pain, fever refractory to antipyretic and antibiotic therapy, and dysphagia (36) have been found as the major causes of hospitalization, and trismus and dysphagia have been considered two important signs of serious infection (36).

It seems therefore indicated to hospitalize all patients in which the infection is refractory to home therapy, and it threatens to spread or has already spread to deep fascial spaces.

No specific indications can be drawn from the present review regarding SI treatment, given the impossibility of correlating the treatment to the severity of the infections and the patient’s clinical conditions. Moreover, the considerable variability of antibiotics used does not allow us to give indications on which is the choice antibiotic, although a targeted approach, based on a culture test, is obviously the option of choice, after an initial empirical antibiotic therapy (37). Incision and drainage allow the pus, which is a potential source of hematogenous spread of the infection, to flow outwards, also preventing its local spread to neighboring regions, especially the deep ones, thus avoiding patient's life risk due to airway compromission (38). Furthermore, decompression of the SIs allows the local symptoms related to the infection to be drastically reduced (39). Antibiotic treatment, on the other hand, can be administered pre-operatively with the sole purpose of reducing the risks of bacteremia and local superinfection related to surgery, especially in immunocompromised patients, while it is necessary for therapeutic purposes when there are clinical signs of systemic compromission due to the infection (40).

## Conclusions

Although lower third molar extraction is the most frequently performed oral surgical procedure, little has been written about post-operative SIs, in relation to risk factors, involved spaces, and performed therapies, so to be able to identify preventive and therapeutic behaviors based on scientific evidence.

Antibiotic prophylaxis was found significantly associated with statistical reduction in the incidence of SIs.

It is not possible to identify, from the available data, whether immunosuppression may or may not represent a risk factor for the occurrence of SIs after third molar extraction.

Likewise, it is not possible to identify whether any anatomical and/or topographical conditions of the lower third molar can be considered predisposing factors as these details cannot be identified from the articles analyzed by the present review.

It is also not possible to identify whether infectious conditions, which affected the third molar at the time of extraction, can represent a risk factor for post-operative SIs.

## Figures and Tables

**Table 1:** Overall data from the selected articles.

Authors	N. of patients	N. of extractions	Suppurative infections N (incidence)	Antibiotic Prophylaxis	Treatment of the infection	Hospitalization after the infection	Ostectomy	N. by Site	Risk factors
Yoshii et al., 2001 (19)	993	993	8 (0.8)	Lenampicillin in all cases	Ampicillin or Cefazolin iv + oral antibiotic therapy after discharge	All cases	NS	6 submandibular space 1 submandibular space + buccal space 1 submandibular space + latero-pharyngeal space.	14 circulatory problems 13 chronic hepatitis 7 diabetes 3 carcinoma 1 rheumatoid arthritis and anemia
Pasupathy et al, 2011 (18)	89	89	5 (5.6)	Amoxicillin 1 gr, before surgery in 31 patients; Metronidazole 800 mg 1 h before surgery in 29 patients; none, in the remain cases	Wound opening and drenaige	None	89	Localized purulent infection	No
Lee et al., 2014 (9)	890	1225	17 (1.3): 14 alveolar abscesses + 3 deep infections	Cefditoren Pivoxil in 439 extraction. No antibiotics in 783 extractions	NS	All cases	NS	14 Alveolar abscess 1 Right sublingual space, 1 Right para-pharyngeal space, 1 Left buccal space	NS
Goldberg et al., 1985 (16)	302	500	21 (4.2)	90 patients. Out of the 21 cases of infection, only 1 received prophylaxis.	16 irrigation + oral antibiotics 4 incision and drainage 1 antibiotic therapy with hospitalization	Only 1 case	NS	NS	Patients on corticosteroid therapy or diabetics were hospitalized, but it was NS how many they were.
Jing et al., 2014 (11)	55	74	2 (2.7)	No	Drainage and antibiotic therapy	No	Yes	NS	None
Piecuch et al., 1995 (12)	2134, including patients who underwent simultaneous extraction of maxillary molars	3443	226 (6.6)	Systemic antibiotics or Tetracyclinpowder in post-extraction cavity, in 3111. None in 332 cases	NS	No	NS	NS	NS
Lacasa et al., 2007 (14)	225	225	18 (8) [placebo group: 12 (16), pre-emptive group: 2 (2.8), antibiotic prophylaxis group: 4 (5.3)]	150 Amoxicillin + Clavulanic acid 75 placebo	NS	No	110 Yes 114 No	NS	None
Artegoitia et al., 2005 (15)	490	490	16 (3,2)	259 Amoxicillin+ Clavulanic acid for 4 days 231 placebo	Metronidazol in Ab group/ Metronidazol + Amoxicillina and Clavulanic acid in the placebo group	No	Yes	Intraoral abscess	None, but 195 patients were smokers
Curran et al., 1974 (13)	68	133	1 (0,75)	33 patients (test group) Pre-op.: Penicillin G, 1 million or Erythromycin, 250 mg i.m. 1 hour before surgery Post-op.: Penicillin or Erythromycin 250 mg, 1 tablet every 6 hours for 4 days	Incision and drainage	Yes	Yes	Intraoral abscess	None
Lloyd et al., 1994 (10)	57	114	3 (2.6)	Metronidazol 400 mg in all cases, 30 patients for 2 days, 27 patients for 3 days	NS	No	Yes	Deep site infection	None
Medhizadeh et al., 2024 (17)	77	77	3 (3.9)	Not performed	NS	No	NS	Sub-periosteal abscess	None

NS = not specified.

**Table 2:** Incidence of suppurative infections (SIs) in relation to antibiotic prophylaxis (AP) and ostectomy by published studies.

Study	AP yes/no		Ostectomy yes/no		Incidence of SIs	
	N° of patients	N° of extractions	N° of patients	N° of extractions	AP yes/no	Ostectomy yes/no
Pasupathy et al. (18)	60 yes, 29 no	60 yes, 29 no	89 yes, 0 no	89 yes, 0 no	2 yes /3 no	5 yes, 0 no
Lacasa et al. (14)	150 yes, 75 no	150 yes, 75 no	110 yes, 115 no	110 yes, 115 no	4% yes (6 pt) / 16% no (12 pt)	12,7% yes (14 pt/ex) 3.5% no (4 pt/ex)
Jing et al. (11)	---------------	0 yes/ 74 no	55 yes/ 0 no	74 yes/ 0 no	0% yes / 2.7% no (2 ex)	2.7% yes (2 ex) / 0 no
Takashi et al. (19)	993 yes / 0 no	993 yes/ 0 no	------------	--------------	0.8% yes (8 pt/ex) / 0 no	----------------
Curran et al. (13)	33 yes / 35 no	------------------	68 yes/ 0 no	133 yes/ 0 no	1 pt, but it is not known to which group he/she belongs	1.4% yes (1 pt) / 0 no (pt)
Goldberg et al. (16)	90 yes / 282 no	--------------	-------------	---------------	1.1% yes (1 pt) / 7.1% no (20 pts)	---------------
Artegoitia et al. (13)	261 yes / 233 no	261 yes / 233 no	494 yes/ 0 no	494 yes/ 0 no	1.9% yes (5 pt/ex) / 4.7 no (11 pt/ex)	3.2% yes (16 pt) / 0% no
Lloyd et al. (10)	57 yes / 0 no	114 yes / 0 no	57 yes / 0 no	114 yes/ 0 no	2.6% yes (3 exs) / 0 no	2.6 % yes (3 ex) / 0 no (ex)
Pieuch et al. (12)	-------------	3089 yes / 332 no	------------	NS	5.14% yes (159 ex) / 14.8% no (49 ex)	NS
Lee et al. (9)	-----------	439 yes/ 783 no	------------	-------------	1.8% yes (8 ex) / 1.14 % no (9 ex)	--------------
Medhizadeh et al. (15)	0 yes / 77 no	0 yes / 77 no	------------	-------------	0%yes / 3.9% no (3 ex)	--------------
TOT	1644 yes/ 731 no (Z = 21.7)	5106 yes/ 1603 no (Z = 2.97)	873 yes/ 115 no (Z = 0.86)	1014 yes/ 115 no (Z = 0.8)	--------------	--------------

AP = antibiotic prophylaxis; NS = not specified; pt = patient(s); ex = extraction(s); Z = zeta test.

**Table 3:** Hospitalization criteria for odontogenic infections.


difficult swallowing and dehydration
risk to the airways or vital spaces
infection spread in moderate and/or high-risk spaces
involvement of the orbital cavities
need for general anesthesia
need for hospital management of present systemic diseases
